# Relationship between epicardial fat volume on cardiac CT and atherosclerosis severity in three-vessel coronary artery disease: a single-center cross-sectional study

**DOI:** 10.1186/s12872-022-02527-7

**Published:** 2022-03-04

**Authors:** Yu Sun, Xiao-gang Li, Kai Xu, Jie Hou, Hong-rui You, Rong-rong Zhang, Miao Qi, Li-bo Zhang, Li-sheng Xu, Stephen E. Greenwald, Ben-qiang Yang

**Affiliations:** 1 College of Medicine and Biological Information Engineering, Northeastern University, Shenyang, People’s Republic of China; 2 Department of Radiology, General Hospital of Northern Theater Command, 83 Wenhua RoadLiaoning Province, Shenyang, 110016 People’s Republic of China; 3 Key Laboratory of Cardiovascular Imaging and Research of Liaoning Province, Shenyang, People’s Republic of China; 4 Department of Cardiology, General Hospital of Northern Theater Command, Shenyang, People’s Republic of China; 5 Blizard Institute, Barts and The London School of Medicine and Dentistry, Queen Mary University of London, London, UK

**Keywords:** Epicardial adipose tissue, Epicardial fat volume, Computed tomography angiography, Three-vessel coronary artery disease

## Abstract

**Background:**

The ideal treatment strategy for stable three-vessel coronary artery disease (CAD) patients are difficult to determine and for patients undergoing conservative treatment, imaging evidence of coronary atherosclerotic severity progression remains limited. Epicardial fat volume (EFV) on coronary CT angiography (CCTA) has been considered to be associated with coronary atherosclerosis. Therefore, this study aims to evaluate the relationship between EFV level and coronary atherosclerosis severity in three-vessel CAD.

**Methods:**

This retrospective study enrolled 252 consecutive patients with three-vessel CAD and 252 normal control group participants who underwent CCTA between January 2018 and December 2019. A semi-automatic method was developed for EFV quantification on CCTA images, standardized by body surface area. Coronary atherosclerosis severity was evaluated and scored by the number of coronary arteries with ≥ 50% stenosis on coronary angiography. Patients were subdivided into groups on the basis of lesion severity: mild (score = 3 vessels, n = 85), moderate (3.5 vessels ≤ score < 4 vessels, n = 82), and severe (4 vessels ≤ score ≤ 7 vessels, n = 85). The independent sample *t*-test, analysis of variance, and logistic regression analysis were used to evaluate the associations between EFV level and severity of coronary atherosclerosis.

**Results:**

Compared with normal controls, three-vessel CAD patients had significantly higher EFV level (65 ± 22 mL/m^2^ vs. 48 ± 19 mL/m^2^; *P* < 0.001). In patients with three-vessel CAD, there was a progressive decline in EFV level as the score of coronary atherosclerosis severity increased, especially in those patients with a body mass index (BMI) ≥ 25 kg/m^2^ (75 ± 21 mL/m^2^ vs. 72 ± 22 mL/m^2^ vs. 62 ± 17 mL/m^2^; *P* < 0.05). Multivariable regression analysis showed that both BMI (*OR* 3.40, 95% CI 2.00–5.78, *P* < 0.001) and the score of coronary atherosclerosis severity (*OR* 0.49, 95% CI 0.26–0.93, *P* < 0.05) were independently related to the change of EFV level.

**Conclusion:**

Three-vessel CAD patients do have higher EFV level than the normal controls. While, there may be an inverse relationship between EFV level and the severity of coronary atherosclerosis in patients with three-vessel CAD.

## Background

Patients suffering from three-vessel coronary artery disease (CAD) often have long-standing and complex coronary atherosclerosis and are at an increased risk of adverse cardiovascular events [1]. Many randomized clinical trials results have revealed no significant differences in survival benefit between the two most widely used treatment, namely percutaneous coronary intervention and coronary artery bypass grafting, for the therapy of multivessel CAD patients [2–4]. Recently, another multicenter randomized trial reported that, in stable CAD, there was no evidence that an initial invasive strategy reduced the risk of adverse cardiovascular events in comparison to medical therapy alone [5]. Therefore, it is difficult to make the best choice of treatment strategy for stable three-vessel CAD patients. For patients who have undergone initial conservative treatment, evaluation of coronary atherosclerotic severity and assessment of its progression are in great demand, as an important aid in deciding further treatment, such as adjustment of drug dose or the ideal timing of invasive strategies.

Epicardial adipose tissue is a type of visceral fat that is located between the surface of the myocardium and the visceral pericardial layer. By secreting a large variety of bioactive molecules, it can modulate vascular inflammation via paracrine signaling mechanisms which, in turn contribute to the development of coronary atherosclerosis and the destabilization of existing atherosclerotic lesions [6]. Many clinical studies have reported that the epicardial fat volume (EFV) using coronary CT angiography (CCTA) is associated with the presence and severity of coronary atherosclerosis and with the characteristics of atherosclerotic plaques [7–9]. Measuring the volume of epicardial fat has been considered as a potential imaging biomarker of disease progression and response to treatment [10]. However, clinical evidence of the relationship between EFV level and coronary atherosclerosis severity in three-vessel CAD patients remains limited, except for that arising from subgroup analysis in previously reported literature [11].

Threrfore, in the present study, we sought to investigate the relationship between EFV level assessed with CCTA and coronary atherosclerosis severity in three-vessel CAD.

## Methods

### Study population

**Fig. 1:**
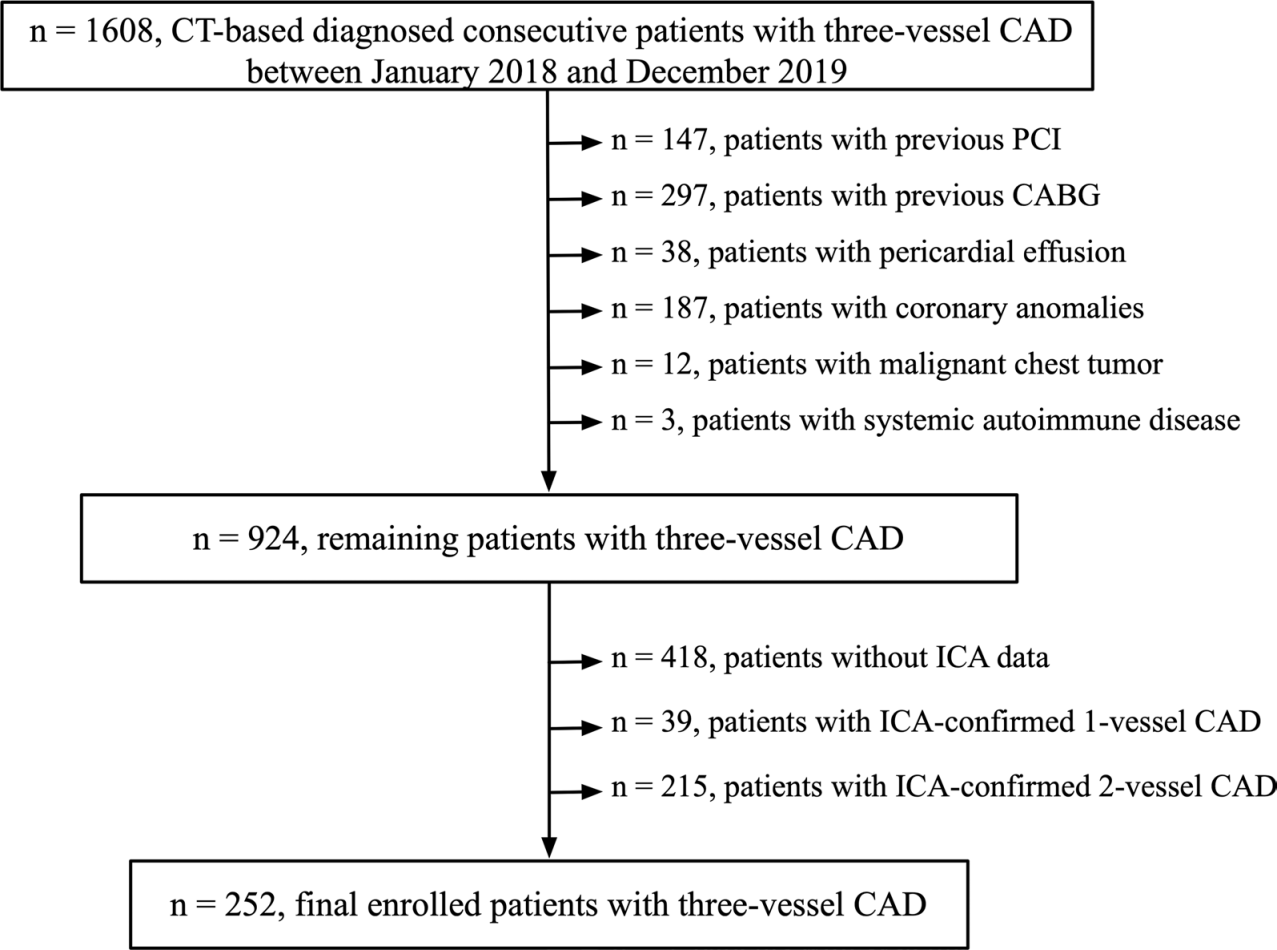
Study flowchart. *CT* computed tomography, *CAD* coronary artery disease, *PCI* percutaneous coronary intervention, *CABD* coronary artery bypass grafting, *ICA* invasive coronary angiography

In this single-center retrospective cross-sectional study, between 1 January 2018 and 31 December 2019, 1608 consecutive patients who underwent CCTA scanning in our hospital and had CCTA-based diagnosis of three-vessel CAD were initially included. Patients with previous percutaneous coronary intervention (n = 147) or coronary artery bypass grafting (n = 297), pericardial effusion (n = 38), coronary anomalies (n = 187), malignant chest tumor (n = 12), or systemic autoimmune disease (n = 3) were excluded. A further 418 patients without invasive coronary angiography (ICA) data were excluded. Patients with ICA-confirmed one- (n = 39) and two-vessel (n = 215) CAD were also excluded. Finally, 252 consecutive patients with ICA-confirmed three-vessel CAD were enrolled (Fig. 1). The time interval between CCTA imaging and ICA operating of all patients was (37 ± 15) days. Three-vessel CAD was defined as ≥ 50% luminal diameter stenosis present in three main epicardial coronary arteries on the ICA data, including the left anterior descending artery, left circumflex, and right coronary artery, with or without the left main artery involvement.

For the normal control group, 252 age-, gender- and body mass index (BMI)-matched subjects were selected. They underwent CCTA because of chest pain from January 2018 to December 2019 and all had negative coronary atherosclerosis and no known cardiovascular disease.

### Cardiovascular risk factors and clinical data

Demographic characteristics and clinical data (summarized in Table 1) were collected for all patients, including BMI, smoking history, hypertension, dyslipidemia, diabetes mellitus and family history of CAD. In addition, measures of left ventricular (LV) function assessed with transthoracic echocardiography were obtained from the hospital records. These included LV end-diastolic diameter, LV ejection fraction (LVEF), and the ratio of diastolic mitral inflow velocity E peak and A peak (E/A). Coronary collateral circulation status and SYNTAX score assessed with ICA data were also collected.

**Table 1:** Basic characteristics and clinical data of study population

Variables	Normal control (n = 252)	Three-vessel CAD patients*			*P-*value
		All patients (n = 252)	Low EFV level (< 118 mL, n = 125)	High EFV level (≥ 118 mL, n = 127)	
EFV (mL)	89 ± 32	120 ± 42	86 ± 21	154 ± 29	< 0.001^#^
EFV/BSA (mL/m^2^)	48 ± 19	65 ± 22	49 ± 12	82 ± 16	< 0.001^#^
Epicardial FAI (HU)	− 84 ± 9	− 81 ± 5	− 78 ± 4	− 84 ± 4	< 0.001^#^
Cardiovascular risk factors					
Age (years)	61 ± 8	61 ± 8	61 ± 9	62 ± 7	0.163
Male sex, n (%)	193 (77)	193 (77)	93 (74)	100 (79)	0.416
BMI (kg/m^2^)	25 ± 3	25 ± 3	24 ± 3	26 ± 3	< 0.001
Smoking, n (%)	37 (19)	125 (50)	60 (48)	65 (51)	0.614
Hypertension, n (%)	0 (0)	179 (71)	85 (68)	94 (74)	0.293
Dyslipidemia, n (%)	0 (0)	45 (19)	21 (17)	24 (19)	0.664
Diabetes, n (%)	0 (0)	104 (41)	48 (38)	56 (44)	0.359
CAD family history, n (%)	0 (0)	29 (12)	17 (14)	12 (9)	0.302
LV functional parameters					
LV diameter (mm)	48 ± 3.8	48 ± 4.3	48 ± 4.5	49 ± 4.0	0.023
LVEF (%)	61 ± 5.0	60 ± 6.0	60 ± 6.4	60 ± 5.6	0.911
E/A	1.6 ± 0.3	1.3 ± 0.4	1.2 ± 0.4	1.3 ± 0.4	0.056
Invasive coronary angiography					
CCC status, n (%)	NA	78 (31)	40 (32)	38 (30)	0.721
SYNTAX score	NA	29 (23, 36)	30 (23, 36)	29 (23, 35)	0.793

*CAD* coronary artery disease, *EFV* epicardial fat volume, *BSA* body surface area, *FAI* fat attenuation index, *BMI* body mass index, *LV* left ventricular, *EF* ejection fraction, *CCC* coronary collateral circulation

*Patients were divided into two subgroups according to median EFV level (118 mL)

^#^*P *values refer to the comparison between three-vessel CAD patients and normal controls. The remaining *P *values refer to comparisons between the low and high EFV level subgroups of the CAD patients

### CCTA data acquisition

CCTA was performed using a 256-slice CT scanner (Brilliance CT, Philips Medical Systems, Cleveland, USA). Detailed scanning parameters were listed as follows: tube voltage, 120 kVp; detector collimation, 128 × 0.625 mm; pitch, 0.16; rotation time, 0.27 s; slice thickness, 0.9 mm; section increment, 0.45 mm. Tube current was set using the ECG-based tube current modulation technique. About 60–70 mL of iodine contrast agent was injected at a rate of 4.5–5.5 mL/s via a high-pressure injector, followed by a 20–30 mL flush of saline at the same rate. A prospective ECG-gated CCTA was triggered using a bolus tracking technique with a trigger threshold of 100 Hounsfield units (HU) in the descending aorta. The mean estimated effective radiation dose of the CCTA scan was (4.6 ± 1.2) mSv.

### EFV level and epicardial FAI measurement

**Fig. 2:**
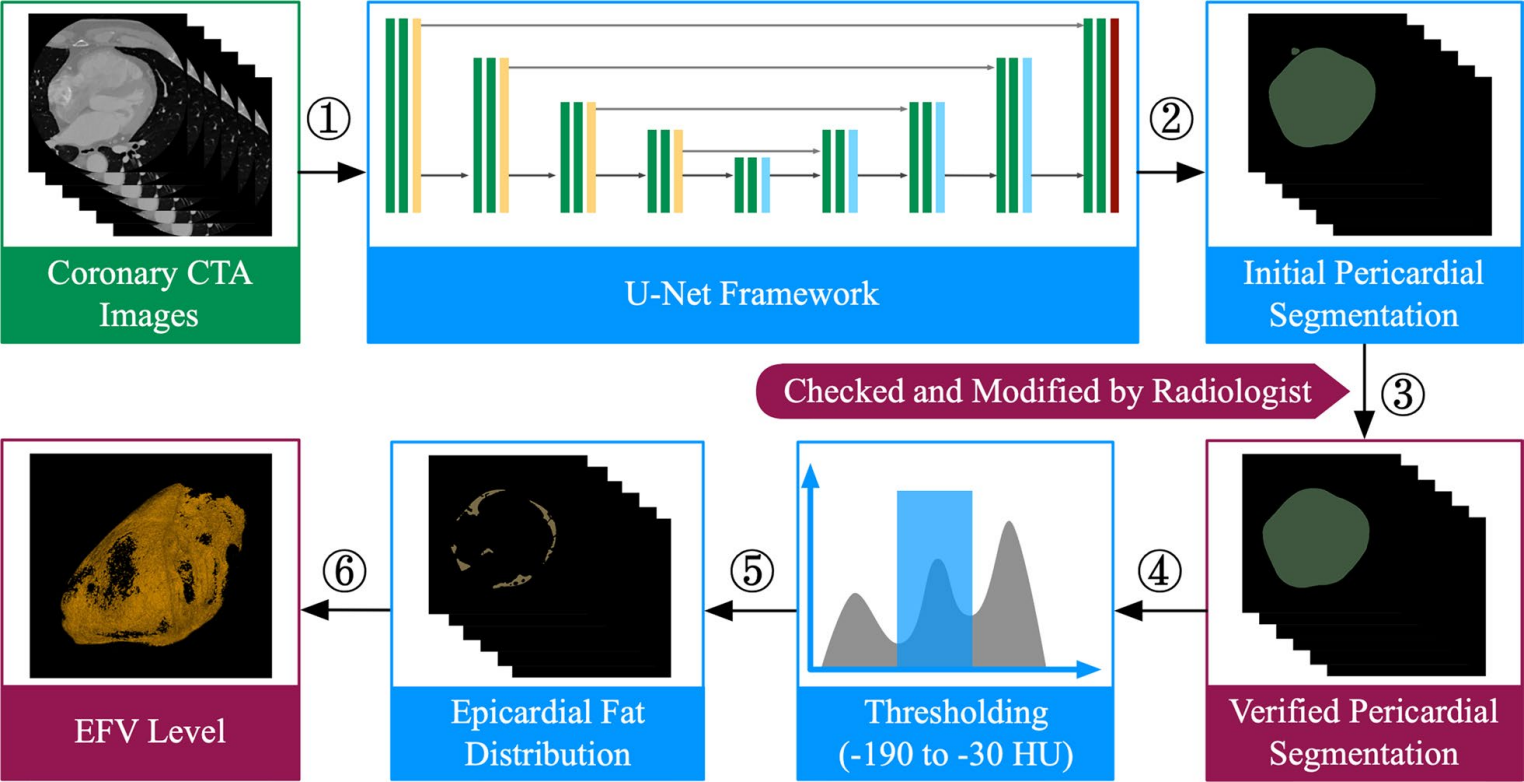
Semi-automatic quantification of EFV level from CCTA images. Axial CCTA images at 75% of the R-R interval were processed for pericardial segmentation using the U-Net framework. The initial results were checked and modified by two radiologists and the epicardial fat was identified as those voxels lying between upper and lower threshold values of − 30 and − 190 HU. EFV epicardial fat volume, CCTA coronary computed tomography angiography

EFV level quantification was quantified on axial CCTA images at 75% of the R-R interval. A semi-automatic method was developed for segmenting the pericardium and measuring the amount of EFV. Briefly, the pericardial contour was automatically delineated by a U-net framework, details of which are described in a previous report from our group [12]. The segmentation results were further checked and modified by two experienced cardiac imaging physicians (J.H. and M.Q.) blinded to both the study plan and the clinical data. Modifications, including manual small scale enlargment or shrinking of the imaging annotation range, were made to ensure that the segmentation results of the pericardial boundary matched exactly with its anatomical structure. The upper boundary of the pericardium was taken as the bifurcation of the pulmonary trunk and the lower limit was the apex of the heart. Epicardial fat was defined as all voxels with attenuation values between − 190 HU and − 30HU. Thus all voxels having attenuation values within this range and lying within the pericardium were thresholded accordingly and used for EFV level quantification (Fig. 2). The value of EFV was normalized to body surface area (BSA) where the BSA was calculated with the Mosteller equation [13]. Epicardial fat attenuation index (FAI) was defined as the average attenuation of adipose tissue within the pre-specified attenuation window of − 190 HU to − 30 HU.

For reproducibility assessment, images from 30 patients with three-vessel CAD were randomly selected. The pericardial contour was automatically delineated using the U-net framework mentioned above. Two radiologists (J.H. and M.Q.) reviewed and modified the automatic pericardial segmentation to ensure a good match with the pericardial anatomy. After four weeks, one of the radiologists (J.H.) reviewed and modified the pericardial segmentation output again. The value of EFV and epicardial FAI derived from the semi-automatic segmentation results of the pericardium was employed for reproducibility assessment using intraclass correlation coefficient analysis.

### Analysis of coronary atherosclerosis severity

Coronary atherosclerosis severity was quantified by the number of coronary arteries having ≥ 50% stenosis on ICA [11, 14]. Stenoses in the left anterior descending, left circumflex, and right coronary artery were each scored as a single vessel lesion and stenoses in the left main coronary artery were more strongly weighted by being scored as two vessel lesions. Stenoses in the remaining secondary branches of the coronary arteries (such as the diagonal artery, obtuse marginal artery, posterolateral artery, and posterior descending artery) with ≥ 50% stenosis were each scored as 0.5 of a lesion.

The three-vessel CAD patients were subdivided into three groups according to coronary atherosclerosis severity: mild (score = 3 vessels, n = 85), moderate (3.5 vessels ≤ score < 4 vessels, n = 82), and severe (4 vessels ≤ score ≤ 7 vessels, n = 85). Patients with more than three vessel lesions were divided into two subgroups based on the median score (4 lesions) of coronary atherosclerosis severity.

### Statistical analysis

Continuous variables were expressed as mean ± standard deviation or medians and interquartile range. Categorical variables were expressed as number and percentage. The Kolmogorov–Smirnov test was employed to test for the normal distribution of continuous data. Comparisons of continuous variables were conducted using the independent *t*-test and one-way analysis of variance (ANOVA), with the least significant difference (LSD) method for multiple comparisons. Comparisons of categorical variables were performed with the Chi-square test. The relation of EFV level to mean epicardial FAI, LV function, and BMI were assessed with Pearson’s correlation analysis. Multivariable logistic regression analysis was used for co-variables that had a significant effect in the univariable logistic regression analysis, aiming to identify the independent factors associated with a change of EFV level. Interobserver and intraobserver agreement for EFV level and epicardial FAI quantification were assessed by evaluating the intraclass correlation coefficient (ICC). Statistical analysis was performed with SPSS software (SPSS statistics, version 26.0, IBM Corp.). Two-sided testing was used and *P* < 0.05 was considered to be statistically significant.

## Results

### Study population characteristics

A total of 252 consecutive three-vessel CAD patients and 252 control group participants were included in this retrospective study. Detailed baseline demographic and clinical data are listed in Table 1. Compared with lesion-free controls, three-vessel CAD patients had significantly higher EFV level (EFV: 120 ± 42 vs 89 ± 32 mL, EFV/BSA: 65 ± 22 vs 48 ± 19 mL/m^2^; *P* < 0.001) and higher mean epicardial FAI (− 81 ± 5 vs − 84 ± 9 HU, *P* < 0.001).

According to the median EFV level (118 cm^3^), three-vessel CAD patients were categorized into low (< 118 mL, n = 125) and high EFV level subgroups (≥ 118 mL, n = 127). Compared with the low EFV subgroup, patients with high EFV had significantly higher BMI (26 ± 3 vs 24 ± 3 kg/m^2^, *P* < 0.001) and larger LV diameter (49 ± 4.0 vs 48 ± 4.5 mm, *P* = 0.023) (Table 1).

### Relation of EFV level to risk factors and LV function

Subgroup analysis of EFV level, separated into two groups by median BMI (25 kg/m^2^), revealed a significantly higher EFV in overweight patients than in those with normal body weight (EFV: 134 ± 41 vs 105 ± 39 mL, *t* = 5.786, *P* < 0.001; EFV/BSA: 70 ± 21 vs 60 ± 21 mL/m^2^, *t* = 3.603, *P* < 0.001). Hypertensive patients had a significantly higher EFV (123 ± 42 vs 110 ± 42 mL, *t* = 2.229, *P* = 0.027; EFV/BSA: 67 ± 21 vs 60 ± 22 mL/m^2^, *t* = 2.322, *P* = 0.021). In comparison to patients who had normal LV diastolic function (E/A > 1, n = 203), those with LV diastolic dysfunction (E/A < 1, n = 49) had a lower EF and lower EFV relative to BSA, although only the latter was statistically significant (EFV: 110 ± 43 vs 122 ± 42 mL, *t* = − 1.868, *P* = 0.063; EFV/BSA: 60 ± 22 vs 67 ± 21 mL/m^2^, *t* = − 1.988, *P* = 0.048). However, there were no significant differences of EFV level between the two subgroups in the variables related to gender, smoking, dyslipidemia, diabetes mellitus, or family history of CAD (*P* > 0.05).

Pearson correlation analysis showed that, for all CAD subjects, EFV was positively and significantly correlated with BMI (*r* = 0.407, *P* < 0.001) and negatively, with mean epicardial FAI (*r* = − 0.709, *P* < 0.001).

### Relation of EFV, FAI, and LV function to the severity of coronary atherosclerosis

**Fig. 3:**
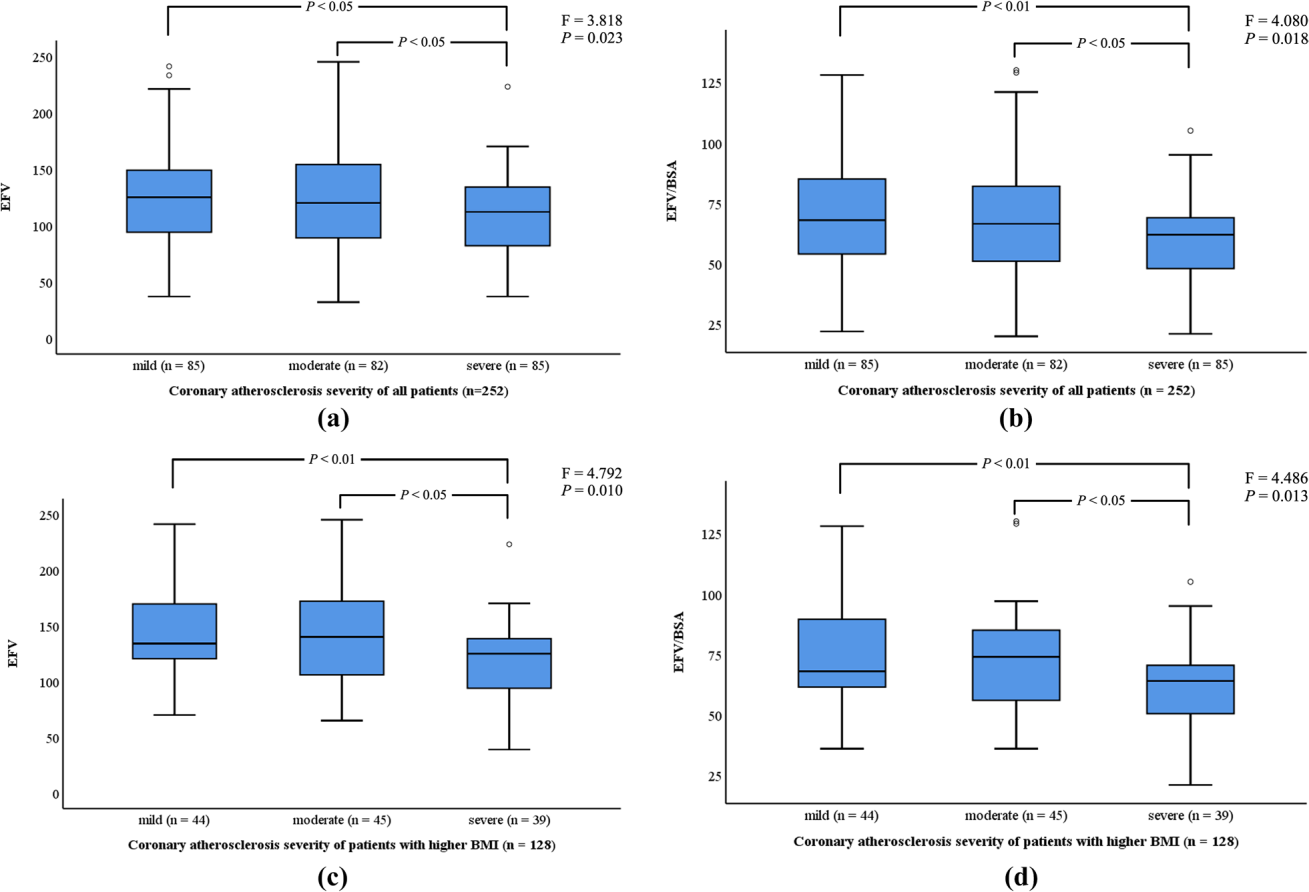
Relation of EFV level to coronary atherosclerosis severity in three-vessel CAD. Three-vessel CAD patients with more severe coronary atherosclerosis have significantly lower EFV level (**a**, **b**), especially in those patients with higher BMI (≥ 25 kg/m^2^) (**c**, **d**). EFV, epicardial fat volume; CAD, coronary artery disease; BMI, body mass index.

Table 2 and Fig. 3a, b show that increasing stenosis severity was significantly associated with reduced EFV. There was a statistically significant inverse relationship between EFV and severity of coronary atherosclerosis (126 ± 45 vs 124 ± 45 vs 110 ± 34 mL, F = 3.818, *P* = 0.023). Post hoc tests using the LSD method showed that the differences between the mild and severe coronary atherosclerosis subgroups were statistically significant (*t* = 16.212, *P* = 0.012), as well as for the comparison between the moderate and severe subgroups (*t* = 14.301, *P* = 0.028). When normalized to BSA, the relationship was maintained and remained significant although the effect was weaker (69 ± 23 vs 67 ± 23 vs 60 ± 17 mL/m^2^, *P* = 0.036).

**Table 2:** Comparison of EFV level, FAI, and LV function in three-vessel CAD

Variables	Score of coronary atherosclerosis severity*			*P-*value
	Mild	Moderate	Severe	
All patients (n = 252)	85 (34)	82 (32)	85 (34)	
EFV (mL)	126 ± 45	124 ± 45	110 ± 34	0.045
EFV/BSA (mL/m^2^)	69 ± 23	67 ± 23	60 ± 17	0.036
Epicardial FAI (HU)	− 81 ± 5.5	− 77 ± 4.5	− 73 ± 4.3	< 0.001
LV diameter (mm)	48 ± 4.3	48 ± 4.5	48 ± 4.0	0.967
LVEF (%)	60 ± 6.3	60 ± 5.6	60 ± 6.2	0.952
E/A	1.3 ± 0.4	1.3 ± 0.3	1.3 ± 0.4	0.931
Patients stratified by BMI^#^				
BMI < 25 kg/m^2^ (n = 124)	41 (33)	37 (30)	46 (37)	
EFV (mL)	107 ± 43	107 ± 42	103 ± 31	0.86
EFV/BSA (mL/m^2^)	62 ± 24	62 ± 24	58 ± 17	0.857
Epicardial FAI (HU)	− 79 ± 6.6	− 77 ± 4.7	− 72 ± 3.9	< 0.001
LV diameter (mm)	48 ± 4.3	47 ± 4.3	48 ± 4.0	0.448
LVEF (%)	60 ± 5.8	61 ± 6.1	60 ± 6.2	0.788
E/A	1.2 ± 0.5	1.3 ± 0.3	1.2 ± 0.3	0.945
BMI ≥ 25 kg/m^2^ (n = 128)	44 (34)	45 (35)	39 (31)	
EFV (mL)	144 ± 40	139 ± 43	118 ± 35	0.01
EFV/BSA (mL/m^2^)	75 ± 21	72 ± 22	62 ± 17	0.013
Epicardial FAI (HU)	− 82 ± 4.0	− 78 ± 4.4	− 74 ± 4.5	< 0.001
LV diameter (mm)	49 ± 4.2	49 ± 4.4	49 ± 4.2	0.802
LVEF (%)	60 ± 6.7	60 ± 5.2	59 ± 6.2	0.725
E/A	1.3 ± 0.4	1.3 ± 0.3	1.3 ± 0.4	0.825

*EFV* epicardial fat volume, *FAI* fat attenuation index, *LV* left ventricular, *CAD* coronary artery disease, *BSA* body surface area, *EF* ejection fraction, *BMI* body mass index

*Patients were subdivided into groups according to their coronary atherosclerosis severity: mild (score = 3 vessels), moderate (3.5 ≤ score < 4 vessels), and severe (4 ≤ score ≤ 7 vessels)

^#^Patients were categorized by median BMI (25 kg/m^2^)

When grouped according to the median BMI (25 kg/m^2^), there was, similarly, a progressive decline in EFV level from the mild, moderate to severe coronary atherosclerosis subgroups in the higher BMI group (EFV: 144 ± 40 vs 139 ± 43 vs 118 ± 35 mL, *P* = 0.01; EFV/BSA: 75 ± 21 vs 72 ± 22 vs 62 ± 17 mL/m^2^, *P* = 0.013) (Table 2; Fig. 3c, d). Post hoc tests using the LSD method showed that, in comparison to the severe atherosclerosis subgroup, both the mild (EFV: *t* = 25.678, *P* = 0.004; EFV/BSA: *t* = 12.670, *P* = 0.005) and the moderate atherosclerosis subgroups (EFV: *t* = 20.480, *P* = 0.020; EFV/BSA: *t* = 10.164, *P* = 0.023) had significantly higher EFVs. However, no significant relationship between EFV level and atheroslcerotic severity was seen in patients with BMIs below the median value Additionally, there was a significant positive association between the severity of coronary atherosclerosis and the mean attenuation due to epicardial fat (FAI) (− 81 ± 5.5 vs − 77 ± 4.5 vs − 73 ± 4.3 HU, *P* < 0.001) for the mild, moderate and severe atherosclerosis levels, respectively. However, there were no significant differences between the mild, moderate, and severe atherosclerosis subgroups in the variables related to LV function, namely LV diameter, LVEF, or E/A (*P* > 0.05).

### Analysis of factors related to the change of EFV level

Univariate and multivariable logistic regression analysis showed that BMI (*OR* 3.40, 95% CI 2.00–5.78, *P* < 0.001) and coronary atherosclerosis severity (*OR* 0.49, 95% CI 0.26–0.93, *P* = 0.03) were independently associated with the change of EFV level in all patients (Table 3).

**Table 3:** Factors associated with EFV level in three-vessel CAD

Variables	Univariable analysis		Multivariable analysis (univariable *P* < 0.05)	
	OR (95% CI)	*P* value	OR (95% CI)	*P* value
Age	1.02 (0.99; 1.06)	0.163		
Gender	0.70 (0.38; 1.19)	0.168		
Smoking	1.21 (0.74; 1.98)	0.452		
Hypertension	1.45 (0.81; 2.60)	0.211		
Dyslipidemia	1.40 (0.75; 2.63)	0.294		
Diabetes	1.26 (0.76; 2.07)	0.366		
BMI^*^	3.60 (2.14; 6.05)	< 0.001	3.40 (2.00; 5.78)	< 0.001
CAD family history	0.66 (0.30; 1.45)	0.304		
LV diameter	1.07 (1.00; 1.14)	0.025	1.06 (0.99; 1.13)	0.104
LVEF	1.00 (0.96; 1.05)	0.91		
E/A	1.94 (0.98; 3.84)	0.058		
Score of coronary atherosclerosis severity^#^	0.49 (0.27; 0.90)	0.022	0.49 (0.26; 0.93)	0.03
CCC status	0.91 (0.53; 1.55)	0.72		
SYNTAX score	0.99 (0.97; 1.02)	0.57		

*EFV* epicardial fat volume, *CAD* coronary artery disease, *OR* odds ratio, *BMI* body mass index, *LV* left ventricular, *EF* ejection fraction, *CCC* coronary collateral circulation

^*^Patients were divided into lower (< 25 kg/m^2^) and higher (≥ 25 kg/m^2^) BMI groups

^#^Patients were divided into groups according to their coronary atherosclerosis severity: mild (score = 3 vessels), moderate (3.5 ≤ score < 4 vessels), and severe (4 ≤ score ≤ 7 vessels)

### Reproducibility assessment

Intraclass correlation coefficient analysis revealed excellent interobserver and intraobserver agreement for EFV level and epicardial FAI measurement. Interobserver analysis showed 0.97, 0.95 agreement for EFV level and epicardial FAI, respectively. Intraobserver analysis showed 0.99, 0.97 agreement for EFV level and epicardial FAI, respectively.

## Discussion

The results show that three-vessel CAD patients had significantly higher EFV levels than those of the normal controls. Furthermore, stratifying the severity of atherosclerosis into mild, moderate and severe levels showed that there was an inverse relationship between EFV level assessed with CCTA and coronary atherosclerosis severity in three-vessel CAD.

Many clinical studies have demonstrated that EFV level on CCTA is related to the presence and severity of coronary atherosclerosis. For instance, in the Framingham Heart Study, measurements in more than 3000 individuals showed that increased EFV was associated with the presence of CAD [8]. EFV level was also related to coronary atherosclerosis severity, coronary artery calcium score, and the characteristics of unstable atherosclerotic plaques [8–10]. Here, we found that three-vessel CAD patients had significantly higher EFVs than those of the normal controls, which is consistent with the previous studies [8–10].

Interestingly, we observed a progressive decline in EFV level with increasing coronary atherosclerotic severity in all three-vessel CAD patients, although it was statistically significant only those with BMI ≥ 25 kg/m^2^. Gorter et al. [11] also reported that patients with more severe coronary atherosclerosis tended to have lower EFVs although their study sample contained only 128 subjects. We believe there are two plausible explanations for this inverse association between disease severity and EFV. One is that fewer numbers of epicardial adipocytes and an increased number of small and immature epicardial perivascular preadipocytes in patients with severe coronary atherosclerosis would imply a reduced absolute EFV level. In the three-vessel CAD patients with chronic coronary vascular inflammation and severe coronary atherosclerosis, chronic hypoxia and an unfavorable hemodynamic environment would affect the regulatory component of the epicardial fat proteasome, which would accelerate the apoptosis of epicardial adipocytes. This hypothesis is supported by evidence that several genes involved in cellular function are downregulated in the epicardial adipocytes of advanced CAD [15]. Furthermore, recent evidence has revealed a bidirectional signaling between epicardial adipose tissue and vascular inflammatory cells [16, 17]. Epicardial adipocytes could, therefore, modulate structural changes and inflammation in the vascular wall via paracrine mechanisms and vascular wall-derived inflammatory factors could block the differentiation of epicardial adipocytes. In three-vessel CAD patients, the remodelling of adipocytes due to vessel inflammation was more obvious than that in patients with mild or moderate CAD. Therefore, the end result could be large proportion of small and immature epicardial perivascular pre-adipocytes around vessels, thus leading to a reduction of EFV. The second explanation for reduced EFV in those with severe CAD is related to changes in the composition of epicardial adipocytes which could increase the degree to which they attenuate incident x-rays. If this were to happen, fewer voxels would fall within the thresholded range and, this would lead to an apparent reduction in EFV. This effect is likely to be seen in CAD patients who have had long-standing and complex coronary atherosclerosis, which is associated with irreversible changes in epicardial perivascular adipocyte composition, such as inflammatory infiltration, microvascular remodeling and extracellular fibrosis [18, 19]. Furthermore, according to the bidirectional interaction hypothesis mentioned above [16, 17], epicardial fat preadipocytes tend to be of smaller size and contain less intracellular lipid than that of mature epicardial adipocytes. The loss of balance between lipid and water content in epicardial perivascular preadipocytes would result in a rise of epicardial FAI. The change of epicardial fat composition in three-vessel CAD patients with more severe coronary atherosclerosis leads to an increase of epicardial FAI, which, in turn, leads to a significant reduction of EFV level when measuring CCTA images using threshold-based methods. This hypothesis is consistent with the results reported here in which three-vessel CAD patients with severe coronary atherosclerosis had significantly higher epicardial FAI. These observations are also consistent with previous studies which have reported increased epicardial FAI in patients with advanced CAD or acute coronary syndrome [20, 21].

Additionally, we observed a significantly higher EFV in hypertensive subjects and those in the higher BMI group, again in line with previous studies [22, 23]. We also found that in patients with LV diastolic dysfunction there was a tendency towards lower EFV, although this was not statistically significant. A possible explanation is that epicardial fat is associated with both visible epicardial coronary atherosclerosis and coronary microvascular dysfunction, both of which would lead to impaired myocardial perfusion and contractile function [24].

There are several limitations to the study. First, it was a retrospective single-center study and the sample size was relatively small. Second, even though we adopted a commonly used method for classifying the severity of coronary atherosclerosis [11, 14], it did not account for the stability of the atherosclerotic plaque. Third, it has been reported that statin therapy is associated with a reduction in epicardial fat accumulation, especially when given in high doses (atorvastatin 80 mg/day) [25]. However, in this study, all the CAD patients were given moderate doses (atorvastatin 10–20 mg/day), this being based on expert recommendation for Chinese subjects [26]. Whether a significant reduction of EFV in three-vessel CAD patients with severe coronary atherosclerosis is associated with moderate statin therapy needs further clinical studies to confirm.

## Conclusions

Patients with three-vessel CAD have significantly higher EFV level than age-, gender- and BMI-matched normal controls. Furthermore, our study results show that there is an potential inverse relationship between EFV level and coronary atherosclerosis severity in three-vessel CAD. Therefore, the value of clinical applications of the current study may be that a fall in EFV level on CCTA images during follow-up of patients with three-vessel CAD is a potential imaging biomarker of disease progression, which will contribute to the clinical management of three-vessel CAD. However, future larger-scale studies are required for the validation of our findings.

## Data Availability

The datasets used and/or analysed during the current study are available from the corresponding author on reasonable request.

## Abbreviations

BMI: Body mass index
BSA: Body surface area
CAD: Coronary artery disease
CCTA: Coronary computed tomography angiography
EF: Ejection fraction
EFV: Epicardial fat volume
FAI: Fat attenuation index
HU: Hounsfield units
ICA: Invasive coronary angiography
LV: Left ventricular
